# Horizontal transfer of a eukaryotic plastid-targeted protein gene to cyanobacteria

**DOI:** 10.1186/1741-7007-5-26

**Published:** 2007-06-20

**Authors:** Matthew B Rogers, Nicola J Patron, Patrick J Keeling

**Affiliations:** 1 Canadian Institute for Advanced Research, Department of Botany, University of British Columbia, 3529-6270 University Boulevard, Vancouver, BC V6T 1Z4, Canada

## Abstract

**Background:**

Horizontal or lateral transfer of genetic material between distantly related prokaryotes has been shown to play a major role in the evolution of bacterial and archaeal genomes, but exchange of genes between prokaryotes and eukaryotes is not as well understood. In particular, gene flow from eukaryotes to prokaryotes is rarely documented with strong support, which is unusual since prokaryotic genomes appear to readily accept foreign genes.

**Results:**

Here, we show that abundant marine cyanobacteria in the related genera *Synechococcus *and *Prochlorococcus *acquired a key Calvin cycle/glycolytic enzyme from a eukaryote. Two non-homologous forms of fructose bisphosphate aldolase (FBA) are characteristic of eukaryotes and prokaryotes respectively. However, a eukaryotic gene has been inserted immediately upstream of the ancestral prokaryotic gene in several strains (ecotypes) of *Synechococcus *and *Prochlorococcus*. In one lineage this new gene has replaced the ancestral gene altogether. The eukaryotic gene is most closely related to the plastid-targeted FBA from red algae. This eukaryotic-type FBA once replaced the plastid/cyanobacterial type in photosynthetic eukaryotes, hinting at a possible functional advantage in Calvin cycle reactions. The strains that now possess this eukaryotic FBA are scattered across the tree of *Synechococcus *and *Prochlorococcus*, perhaps because the gene has been transferred multiple times among cyanobacteria, or more likely because it has been selectively retained only in certain lineages.

**Conclusion:**

A gene for plastid-targeted FBA has been transferred from red algae to cyanobacteria, where it has inserted itself beside its non-homologous, functional analogue. Its current distribution in *Prochlorococcus *and *Synechococcus *is punctate, suggesting a complex history since its introduction to this group.

## Background

Comparative genomics has generated a large pool of molecular data, and one of the major debates to emerge from this data is the importance of horizontal gene transfer in genomic evolution [1]. Cases of prokaryote to prokaryote and prokaryote to eukaryote transfers are manifold, and their importance has dominated this debate [2-4]. However, relatively few cases of eukaryote to prokaryote transfers have been reported, and many are not supported by broad representation or strong phylogenetic results [4]. The apparent paucity of such events in relation to transfers between prokaryotes could reflect biological limitations on gene transfer from eukaryotes to prokaryotes. Such limitations might include the presence of spliceosomal introns, or differences in promoter structures and ribosomal binding sites. Another important consideration, however, is that prokaryotes are far more abundant than eukaryotes in most microbial ecosystems, so there might be limited opportunities for such transfers to occur. Nevertheless, a small number of well-supported examples of eukaryote to prokaryote gene transfer are known. One of the most intriguing of these is the transfer of eukaryotic alpha- and beta-tubulin subunits to the bacterium *Prosthecobacter *[5,6]. Other examples are described in the recent analysis of eukaryotic shikimate pathway enzymes [7]. This study has revealed a possible eukaryote to prokaryote transfer in the gene encoding a class II 3-deoxy-D-arabino-heptulosonate 7-phosphate (DAHP)synthase. Another proposed eukaryote to prokaryote transfer might have occurred in the spirochaete *Treponema pallidum*, which contains a eukaryotic-type GAPDH that is phylogenetically related to euglenozoan homologues [8]. Here, we report another example of eukaryote to prokaryote gene transfer with a number of unique functional and evolutionary implications.

Fructose bisphosphate aldolase (FBA, or aldolase) is a core carbon metabolic enzyme responsible for the aldol cleavage of fructose-bisphosphate into glyceraldehyde-3-phosphate (GAP) and dihydroxyacetone phosphate (DHAP) sugars in glycolytic reactions, and the reverse aldol condensation of these triose sugars to fructose-bisphosphate in Calvin cycle and gluconeogenic reactions. FBA exists in two non-homologous but functionally equivalent forms, referred to as class I and class II [9]. Eukaryotes typically use class I FBA, although there are a few noteworthy exceptions [10-13]. In contrast, prokaryotes typically use the class II form of the enzyme, although once again a handful of species also encode a class I form of unknown function. One other interesting exception is found in the plastid (chloroplast) of plants and many algae. A large number of cyanobacterial genomes have been shown to encode a canonical, bacterial class II FBA, indicating that this FBA is ancestral to the group, as one would expect for prokaryotes. As the descendants of endosymbiotic cyanobacteria, plastids would also be expected to contain this cyanobacterial class II FBA, but this enzyme has been replaced by a duplicate of the host cytoplasmic class I enzyme in the ancestors of plants, red and green algae, resulting in two class I enzymes in these groups [14]. Hence, glaucophyte plastids are unique in having retained their ancestral protein [12].

Here we show that this eukaryotic plastid-targeted class I FBA has itself been transferred to a specific group of marine cyanobacteria. The complete genomes of several strains of both *Prochlorococcus *and *Synechococcus *encode a class I FBA gene. We show that these are evolutionarily related to the gene for the plastid-targeted protein of red algae. In some strains, the ancestral class II gene has also been lost, suggesting that the eukaryotic gene has completely taken over its function. The position of the transferred gene is also of interest, as it is immediately upstream of the class II FBA in genomes where both are present. In genomes where the class II FBA has been lost, the new class I gene therefore occupies the position previously occupied by class II FBA.

## Results and Discussion

### Class I and class II FBA in *Prochlorococcus *and *Synechococcus*

**Figure 1:**
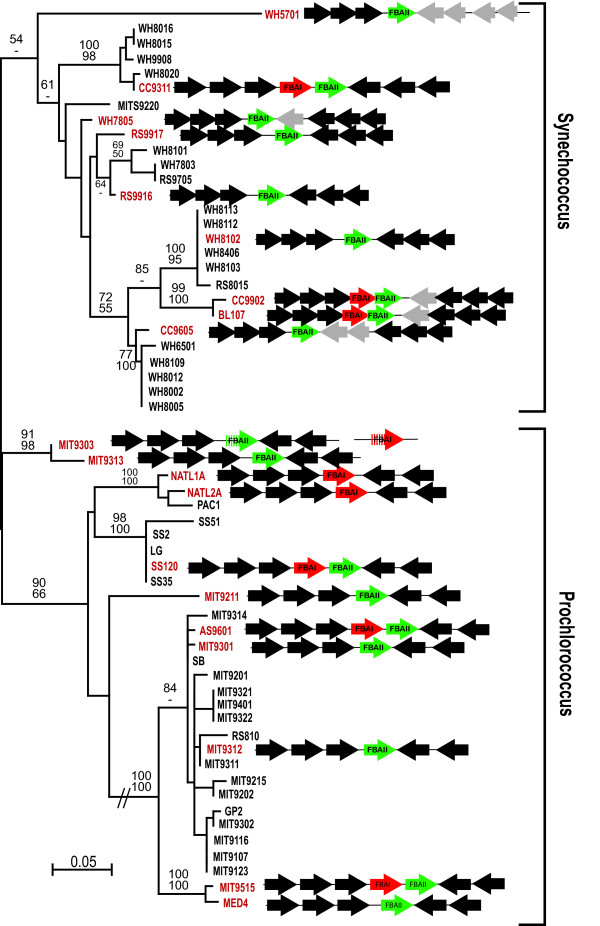
**ITS Phylogeny of *Prochlorococcus *and *Synechococcus *showing gene order surrounding FBA I locus**. Phylogeny of *Prochlorococcus *and *Synechococcus *strains showing the distribution of class I and class II FBA. Maximum likelihood tree based on rRNA ITS sequences with bootstrap support shown for major nodes with greater than 70% support. The branch leading to one clade of *Prochlorococcus *(indicated by double hatch marks) has been truncated to fit. The genomic context of FBA genes is shown for completely sequenced genomes. Class I FBA (eukaryotic) is shown in red and class II (prokaryotic) is shown in green. Black arrows correspond to up and downstream genes that are conserved in order and direction in most genomes, whereas grey arrows are the few exceptions to this conservation.

Cyanobacterial genomes typically encode a class II FBA for use in both glycolysis and the Calvin cycle. However, in two closely related genera, *Synechococcus *and *Prochlorococcus*, we have found the situation is more complex. The order of genes surrounding the FBA locus is highly conserved among completely sequenced representative genomes from both genera. While many strains encode only the ancestral cyanobacterial class II FBA, in three *Synechococcus *strains (BL107, CC9902, and 9311) and three *Prochlorococcus *strains (SS120, AS9601, and MIT9515), a eukaryote-derived class I FBA is found immediately upstream of the ancestral cyanobacterial gene (Figure 1). Moreover, in two *Prochlorococcus *strains (NATL1A and NATL2A) the cyanobacterial class II FBA has been lost, effectively leaving the eukaryotic class I FBA in its place (Figure 1). *Prochlorococcus *MIT9303 also has a homologue of this gene elsewhere in the genome, but both it and the canonical class II FBA contain several stop codons, indicating either they are pseudogenes or sequencing errors.

To compare the distribution of FBA genes to the phylogeny of the organisms, a phylogeny of *Prochlorococcus *and *Synechococcus *strains was constructed using a locus independent of FBA, the 16S-23S internal transcribed spacer (ITS). This phylogeny (Figure 1) largely agrees with previously published analyses [15], with a distinction between the two genera and a further subdivision between high-light and low-light adapted strains of *Prochlorococcus *[15]. However, the phylogeny is incompatible with any simple explanation for the distribution of FBA genes. Most importantly, the organisms that possess the eukaryotic class I FBA do not form a unique clade in the ITS phylogeny, but are instead scattered across the tree. This is exemplified by two pairs of *Prochlorococcus *strains (MIT9301/AS9601 and MED4/MIT9515) that share virtually identical ITS sequences, and are thus inferred to be closely related, but in both cases the former strain encodes only the ancestral class II FBA whereas the latter encodes both classes. The two *Prochlorococcus *strains that encode only the eukaryotic class I gene (NATL1A and NATL2A) are closely related in ITS phylogeny, suggesting the class II FBA was lost once in their common ancestor.

### Origin of class I FBA in *Synechococcus *and *Prochlorococcus*

**Figure 2:**
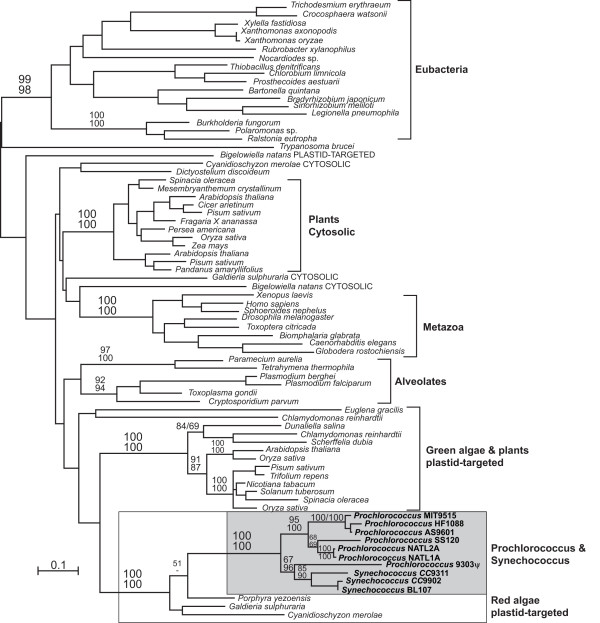
**Protein maximum likelihood tree of class I FBA**. Protein maximum likelihood phylogeny of class I FBA. The cyanobacterial and plastid-targeted red algal class I FBA genes are indicated by boxes, and all other groups are bracketed and labelled to the right. Numbers at node correspond to bootstrap support over 50% for major nodes from ML (above) and distance (below). Methods and parameters used are detailed in the Methods section.

The relatively restricted distribution of this eukaryotic gene in a few strains of *Synechococcus *and *Prochlorococcus *suggests either a very recent origin in these genera, or horizontal gene transfer between strains. To determine if the genes originated once in cyanobacteria and from what kind of eukaryote they might be derived, phylogenetic analyses were performed to determine the position of the cyanobacterial genes relative to eukaryotic class I FBAs. Class I FBA phylogeny has been shown previously to lack sufficient resolution between many major subgroups [14,16], and the present analysis was no exception to this (Figure 2). However, the cyanobacterial genes formed a unique clade with 100% support, indicating a single origin of the gene in *Synechococcus *and *Prochlorococcus*. Most importantly, the cyanobacterial genes formed a specific and strongly-supported group with the nuclear-encoded, plastid-targeted FBAs from red algae (100% support). These genes are very distantly related to the discrete clade of class I FBAs already known from some prokaryotes, which is also present in three cyanobacteria *Trichodesmium erythraeum*, *Crocosphaera watsonii*, and *Synechococystis *PCC6803. These genera are not closely related to *Prochlorococcus *and *Synechococcus*. The *Prochlorococcus *and *Synechococcus *genes show elevated rates of evolution, but with the exception of the possible pseudogene in *Prochlorococcus *MIT9303, none show any signs of being non-functional or otherwise unusual.

An affinity between genes for plastid-targeted proteins and cyanobacterial homologues is generally not surprising. However, this result is unusual in that the red and green algal plastid FBAs are not derived from the cyanobacterial endosymbiont. Instead, these genes have been inferred to result from a duplication of and replacement by the non-homologous, cytosolic class I FBA [14]. Altogether, this eukaryotic class I FBA has invaded a prokaryotic, photosynthetic environment twice: originally invading the endosymbiotic plastid in the ancestor of red and green algae (glaucophytes being the only primary algae that retain the ancestral class II FBA in their plastid [11,12]), and subsequently invading the cyanobacterial lineage itself in the genera *Synechococcus *and *Prochlorococcus*.

### FBA distribution within *Synechococcus *and *Prochlorococcus*

The phylogeny of class I FBA shows the *Prochlorococcus*/*Synechococcus *genes originated once, and strongly suggests this was by horizontal gene transfer from a red alga, specifically involving the gene for its plastid-targeted FBA. However, such an event would lead to a simple distribution of class I FBA in cyanobacteria where all taxa possessing the new gene were closely related to one another. This contrasts with the observed distribution shown in Figure 1, and is best exemplified by *Prochlorococcus *strains AS9601/MIT9301 and MIT9515/MED4, where apparently close relatives differ in the presence or absence of class I FBA. The complexity of this distribution suggests the evolution of class I FBA within *Prochlorococcus *and *Synechococcus *has been characterised either by further gene transfer events, or selective loss and retention of this eukaryotic gene across different strains. These two alternatives are described in greater detail below.

**Figure 3:**
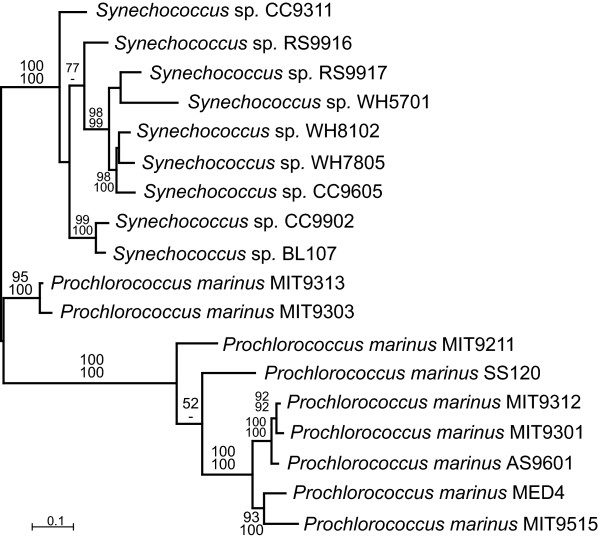
**PHYML tree of class II FBA**. Maximum likelihood phylogeny of *Prochlorococcus *and *Synechococcus *class II FBA using nucleic acids. Numbers at node correspond to bootstrap support over 50% for major nodes from ML (above) and distance (below). Methods and parameters used are detailed in the Methods section.

On the one hand, it is possible that the class I FBA was transferred relatively recently from a red alga to one member of the *Prochlorococcus*/*Synechococcus *clade, and subsequently moved to other strains to achieve its present distribution. While multiple transfer events appears complex, there are well-known precedents for gene transfer between *Prochlorococcus *strains, facilitated by cross-infecting phage [17-19]. This leads to the possibility that the phylogeny of class I FBA might not match that of the strains in which it is found. However, the topology of the *Prochlorococcus*/*Synechococcus *clade in the class I FBA tree (Figure 2) does not differ in any strongly-supported node from the phylogeny inferred from ITS (Figure 1). Moreover, the fact that class I FBA is always found in the same genomic context suggests that such inter-strain transfer would most likely have involved insertion by homologous recombination. This would lead to the additional expectation that the flanking genes might share a greater degree of similarity to homologues in other strains with class I FBA than they do to homologues from strains that lack it. To test this, we inferred phylogenies of the ancestral class II FBA (Figure 3) and genes upstream and downstream of both FBA genes (*mviM *upstream and *purQ *downstream: see Additional file 1). In all three cases the phylogenies mirror the ITS phylogeny for strongly-supported nodes relative to the distribution of FBA genes, and in no case do they reveal a clade consisting of the class I FBA-containing strains. These gene sequences were also manually examined for evidence of small-scale recombination at the ends proximal to FBA, and none was observed. There is therefore no evidence that the evolutionary history of either class I or class II FBA has recently deviated from that of the organisms in which they are found (inferred from ITS phylogeny).

As there is no direct evidence for recombination in this region between strains containing class I FBA, and no indication that the evolutionary history of *Prochlorococcus *and *Synechococcus *class I FBA genes differs from the organisms in which they are found, there is no reason to believe the gene has moved between strains. A more likely explanation is that class I FBA was acquired in the ancestor of *Prochlorococcus *and *Synechococcus *and its current distribution is due to differential gene loss and retention events. As with the horizontal transfer explanation, there is also a precedent for aldolase loss events in these genera, as the ancestral class II FBA has clearly been lost in *Prochlorococcus *NATL1A and NATL2A. This explanation is consistent with all phylogenetic data, but without more sampling from additional strains, it would be premature to definitively distinguish between these two alternatives, or some combination of both.

### Implications for FBA evolution and function

The existence and the punctate distribution of the eukaryotic class I FBA in *Prochlorococcus *and *Synechococcus *both have interesting implications for the evolution and function of this enzyme in cyanobacteria.

A single early origin of the eukaryotic class I FBA in *Prochlorococcus *and *Synechococcus *implies that both genes were present in their common ancestor and that redundancy was common throughout their evolution. The eukaryotic enzyme would not be retained without having acquired some function, which is most clearly demonstrated in the NATL1A and NATL2A strains where the ancestral class II gene has been lost. The maintenance of both types of FBA in several strains today, particularly in the minimal genome of *Prochlorococcus *SS120 [20], further supports the conclusion that the class I FBA has some function. It is possible that the two classes of FBA are functionally differentiated, perhaps between glycolysis and the Calvin cycle, or perhaps one recognised a new substrate (as might also be the case with the other discrete clade of bacterial class I FBA genes in Figure 2). Any such differentiation must be reversible, however, as both classes have been lost at least once. If we consider only nodes with greater than 90% support from at least one method in Figure 1, then the eukaryotic class I FBA would have to have been lost at least once in *Synechococcus *and at least three times in *Prochlorococcus*. In reality these figures are likely larger, because many nodes in the phylogeny that are not taken into account are also consistently recovered and well supported in the other phylogenies. The ancestral class II gene would also have to have been lost at least once in the ancestor of *Prochlorococcus *NATL1A and NATL2A, which has remarkable implications for the function of this enzyme in the Calvin cycle. Indeed, if any of the gene losses implied by the distribution of FBA genes led to its function being assumed or re-assumed by its analogue, then a closer examination of the biochemical activities of class I and class II FBA in strains of *Prochlorococcus *and *Synechococcus *where both types of FBA are present will be interesting. Further examination might reveal conditions under which each class of FBA is favoured, addressing the question of why the eukaryotic protein was retained at all.

These considerations also need to take into account the unusual position of the eukaryotic gene in the genome. Its situation immediately upstream of its analogue might be chance, but this would be extremely fortuitous. We suggested above that this position might have favoured the retention of the new gene because the regulation of this position might have been favourable for a new FBA gene due to the presence of the existing one. However, if these two genes are part of an operon, functional differentiation might have taken place without expression-level differentiation. For example, temporal differentiation (such as light/dark cycles) could easily lead to the two enzymes adapting to different roles, but if they are co-expressed such differentiation becomes more difficult. This once again points to the importance of re-evaluating the functional differences between these two classes of FBA to see if there is some mechanistic property that might convey a selective advantage of class I FBA over class II, perhaps only manifested in one direction.

## Conclusion

Recent and well-defined cases of horizontal gene transfer from eukaryotes to prokaryotes remain rare, despite the abundance of data from prokaryotic genomes. This example stands out not only as one of the more strongly-supported cases, but also has a number of other interesting implications. The location of the gene immediately upstream of its non-homologous, functional analogue suggests the genomic context of this insertion was decidedly non-random and points to the importance of context in the adaptation of a newly transferred gene. The punctate distribution of the eukaryotic class I FBA among strains of *Prochlorococcus *and *Synechococcus *also suggests the history of this enzyme was subject to further complications that might shed light on the fate of newly acquired genes in prokaryotic genomes.

One of the major outstanding questions of this study is whether the eukaryotic class I FBA confers any advantage over its analogue, as opposed to simply replacing the analogue to no great consequence. In either case, the functional differences between class I and class II FBA proteins should be re-investigated in greater detail, as the possibility that class I FBAs might be more effective in Calvin cycle reactions has broad implications for the evolution of photosynthesis in prokaryotes and eukaryotes, and for the origin and evolution of plastid organelles.

## Methods

### Identification of FBA genes and genomic context

All complete cyanobacterial genomes (and all prokaryotic genomes in general) were searched for both class I and class II FBA gene sequences using Blastp and Blastx. The red alga-derived class I FBA was found in several strains of *Prochlorococcus *and *Synechococcus *but in no other cyanobacterium and no other prokaryotic genome. The annotated identity of the flanking genes was compared and confirmed by database comparisons. Genomic data from unpublished genomes of *Prochlorococcus *AS9601, MIT9211, MIT9301, MIT9303, MIT9312, MIT9315, NATL1A were kindly provided by ML Coleman and SW Chisholm (Massachusetts Institute of Technology, Department of Civil and Environmental Engineering, 15 Vassar Street Bldg., Cambridge, MA).

### Phylogenetic methods

Protein phylogeny of class I FBA was generated from an alignment of 75 taxa and 312 characters using PhyML 2.4.4 [21] with the WAG substitution matrix, four gamma categories and one category of invariable sites. All substitutions matrices were determined using Modelgenerator 0.83 [22]. The gamma shape parameter alpha and the proportion of invariable sites i were estimated by PhyML to be 1.14 and 0.07, respectively. One hundred bootstrap replicates were preformed in the same way, using alpha and i parameters estimated from the original data. Distances were calculated using TREE-PUZZLE v. 5.2 with the WAG substitution matrix, four gamma categories and one category of invariable sites using the same alpha and i estimates. Trees were constructed using WEIGHBOR 1.0.1a and 500 bootstraps carried out using puzzleboot [23]. Nucleotide based phylogenies of ITS, FBA II, *purQ *and *mviM *from *Prochlorococcus *and *Synechococcus *were carried out using the same methods and the same numbers of bootstraps. For ITS (58 taxa and 212 positions), the HKY model of substitution with four gamma categories and one category of invariable sites was used, with the alpha parameter, proportion of invariable sites, and ts:tv ratio estimated from the data (0.40, 0.12, and 3.05, respectively). For class II FBA (18 taxa and 1021 positions), *purQ *(20 taxa and 639 positions) and *mviM *(20 taxa and 1003 positions) the GTR model of substitution with four gamma categories and one category of invariable sites was used. The alpha parameters were estimated to be 2.96, 1.57, and 1.55, and the proportions of invariable sites were estimated to be 0.51, 0.31 and 0.20, for class II FBA, *purQ *and *mviM*, respectively.

## Authors' contributions

MBR, NJP and PJK contributed to the concept of this manuscript. Phylogenetic analyses were performed by MBR and PJK. The original alignment of class I FBAs was provided by NJP. The manuscript was written by MBR and PJK.

## Supplementary Material

Additional file 1 Figure showing phylogenies of *purQ*, *mviM *generated from nucleic acids Numbers at node correspond to bootstrap support over 50% for major nodes from ML (above) and distance (below). Methods and parameters used are detailed in the Methods section. Click here for file
